# Changes in social isolation and loneliness prevalence during the COVID-19 pandemic in Japan: The JACSIS 2020–2021 study

**DOI:** 10.3389/fpubh.2023.1094340

**Published:** 2023-02-16

**Authors:** Hiroshi Murayama, Takumi Suda, Isuzu Nakamoto, Tomohiro Shinozaki, Takahiro Tabuchi

**Affiliations:** ^1^Research Team for Social Participation and Community Health, Tokyo Metropolitan Institute of Gerontology, Tokyo, Japan; ^2^Department of Global Health Research, Juntendo University Graduate School of Medicine, Tokyo, Japan; ^3^Department of Gerontological and Home Healthcare Nursing, Tohoku University Graduate School of Medicine, Miyagi, Japan; ^4^Faculty of Engineering, Tokyo University of Science, Tokyo, Japan; ^5^Cancer Control Center, Osaka International Cancer Institute, Osaka, Japan

**Keywords:** COVID-19, loneliness, social isolation, prevalence, Japan

## Abstract

**Objective:**

The recent coronavirus disease 2019 (COVID-19) outbreak has changed people's social connections with others and society. This study aimed to describe changes in the prevalence of social isolation and loneliness by demographic characteristics, socioeconomic status, health conditions, and outbreak situations in residential prefectures among Japanese people between the first year (2020) and the second year (2021) of the COVID-19 pandemic.

**Methods:**

We used data from the Japan COVID-19 and Society Internet Survey (JACSIS) study, a large-scale web-based nationwide survey conducted with 53,657 participants aged 15–79 years in August–September 2020 and September–October 2021 (25,482 and 28,175 participants, respectively). Social isolation was defined as less than once a week in the total frequency of contact with family members or relatives who were living apart and friends/neighbors. Loneliness was assessed using the three-item University of California, Los Angeles (UCLA) Loneliness Scale (score range, 3–12). We used generalized estimating equations to estimate the prevalence of social isolation and loneliness in each year and the difference in prevalence between 2020 and 2021.

**Results:**

The weighted proportion (95% confidence interval) of social isolation in the total sample was 27.4% (25.9, 28.9) in 2020 and 22.7% (21.9, 23.5) in 2021, representing a change of −4.7 percentage points (−6.3, −3.1). The weighted mean scores of the UCLA Loneliness Scale were 5.03 (4.86, 5.20) in 2020 and 5.86 (5.81, 5.91) in 2021, representing a change of 0.83 points (0.66, 1.00). The detailed trend changes for social isolation and loneliness were noted in the demographic subgroups of socioeconomic status, health conditions, and outbreak situation in the residential prefecture.

**Conclusion:**

Social isolation decreased from the first to the second year of the COVID-19 pandemic, whereas loneliness increased. Assessing the COVID-19 pandemic's impact on social isolation and loneliness contributes to understanding who was particularly vulnerable during the pandemic.

## Introduction

Social isolation is the objective separation from others, and loneliness is the subjective feeling of being alone or separated. Associations between social isolation, loneliness, and various adverse health outcomes have been reported (1–5). Moreover, scholars have argued that the health risks associated with isolation and loneliness are equivalent to the well-established detrimental effects of smoking and obesity (6).

The recent outbreak of the coronavirus disease 2019 (COVID-19) forced people to socially isolate themselves (7) and to feel lonely (8, 9) as a result of strategies enacted in efforts to reduce the risk of infection. This was because people were unable to engage in casual interactions under social distancing and stay-at-home orders (10, 11). One pandemic-related review confirmed that visiting restrictions during the pandemic led to social isolation, loneliness, and negative consequences such as reduced nutritional intake and physical inactivity (12). Several studies have also reported both social isolation (13) and loneliness (14–16) during the pandemic as potential risk factors for poor mental health. Additionally, Murayama et al. reported that those who became socially isolated during the pandemic were likely to experience greater loneliness (7).

Because both social isolation and loneliness had deleterious impacts on people's health conditions not only before but also during the pandemic, the trends in social isolation and loneliness due to the unique context of the COVID-19 pandemic should be carefully monitored. Pedersen et al. investigated time trends in social isolation and loneliness over the first 16 months of the COVID-19 pandemic (between March 2020 and July 2021) in Denmark using nationally representative, repeated cross-sectional data (17). Overall, poorer levels of social isolation and loneliness were observed during the strict lockdown periods, whereas better levels were observed during the reopening phases. Sugaya et al. reported no improvement in social isolation and loneliness between May 2020 and February 2021 among Japanese people, whereas psychological distress and depression significantly decreased (18).

However, this area of research can be improved in at least two ways. First, trends of social isolation and loneliness during the pandemic have not been well-documented because much of the research related to these conditions and experiences was derived from a cross-sectional design. Second, detailed information, such as the changes in people's characteristics and residential environments, is not available, warranting further analysis. This information could contribute to developing effective strategies not only for the current COVID-19 pandemic but also for future pandemics.

Did the COVID-19 pandemic have a negative impact on people's social connections with others and society? To address the gaps mentioned above, this study aimed to describe changes in the prevalence of social isolation and loneliness between the first year (2020) and the second year (2021) of the COVID-19 pandemic among Japanese people using data from large-scale, web-based nationwide surveys. In particular, we compared the prevalence of social isolation and loneliness by demographic characteristics, socioeconomic status, health conditions, and outbreak situations in residential prefectures of Japan.

## Methods

### Study design and participants

Data were obtained from the Japan COVID-19 and Society Internet Survey (JACSIS) study, which is a nationally representative web-based self-reported questionnaire survey. The JACSIS study was performed in 2020 and 2021—the first and second years of the COVID-19 pandemic—using a large internet survey agency (Rakuten Insight, Inc., Tokyo, Japan).

For the 2020 survey, 224,389 panelists aged 15–79 years were invited using random sampling stratified by sex, age, and prefecture (covering all 47 prefectures; Japan is divided into 47 prefectures). The survey was conducted from August 25 to September 30, 2020, after achieving the target number of respondents per gender, age, and prefecture category. This period was determined based on the population distribution in 2019. Consequently, 28,000 individuals responded to the survey. In the 2021 survey, 59,219 panelists were invited, including those who participated in 2020. The second survey period was from September 29 to October 28, 2021, and the newly added panelists were recruited according to stratification of sex, age, and prefecture to have a sample that better represents the regional and national general populations. In total, 31,000 people responded to the survey. Supplementary Figure 1 illustrates the number of new infection cases of COVID-19 reported per day until December 31, 2021, in Japan. The 2020 and 2021 surveys were conducted during the latter half of the second wave and at the end of the fifth wave of the pandemic in Japan.

Responses with discrepancies and/or artificial/unnatural responses were excluded from the analysis to validate data quality. Three categories (providing invalid responses to the items with the instructions “Please choose the second from the bottom,” choosing positive in all sets of questions for drug use, and choosing positive in all sets of questions for the presence of chronic diseases) were used to detect any discrepancies. Consequently, we excluded 2,518 in the 2020 survey and 2,825 in the 2021 survey with discrepant or artificial/unnatural responses (the remaining 25,482 and 28,175 in 2020 and 2021, respectively).

The protocol of the JACSIS study was approved by the Research Ethics Committee of the Osaka International Cancer Institute (approved on June 19, 2020; approval number 20084). Participants were asked to provide web-based informed consent before responding to the online questionnaire, and the option to opt out at any point was provided. The Internet survey agency respected the Protection of Personal Information Act of Japan. After completing the survey, the participants received a credit point known as an “E-point,” which could be used for Internet shopping and cash conversion, as an incentive.

### Measures

#### Social isolation

Social isolation was measured by each participant's frequency of direct and indirect contact with people other than co-residing family members, based on previous studies (19–21). We assessed the frequency of direct and indirect contact using the following eight questions.

- (i) Frequency of meeting your family members or relatives who are living apart.
- (ii) Frequency of contact with family members or relatives who are living apart by email or text messages (e.g., mobile, LINE, Facebook Messenger).
- (iii) Frequency of contact with family members or relatives who are living apart by voice call (e.g., telephone, mobile, LINE, Facebook Messenger, Skype).
- (iv) Frequency of contact with your family members or relatives who are living apart by video call (e.g., LINE, Facebook Messenger, Skype, Zoom).
- (v) Frequency of meeting your friends or neighbors.
- (vi) Frequency of contact with your friends or neighbors by email or text message.
- (vii) Frequency of contact with your friends or neighbors by voice call.
- (viii) Frequency of contact with your friends or neighbors by video call.

The responses included seven options: “almost every day (6–7 times a week),” “4–5 times a week,” “2–3 times a week,” “once a week,” “2–3 times a month,” “once a month,” and “rarely.” The total frequency of contact was calculated based on a previous article (7). We applied the sum of the frequency of contact using the eight questions and regarded contacts less than once a week in the total frequency of contact as social isolation because people who had contacts less than once a week were reportedly more likely to have higher risks of all-cause mortality and onset of dementia and disability (18).

#### Loneliness

Loneliness was assessed using the University of California, Los Angeles (UCLA) Loneliness Scale version 3, Short Form three-item (hereafter the UCLA Loneliness Scale) (22, 23). In the JACSIS study, we used the Japanese version of the scale, whose validity and reliability had already been confirmed (24). The original UCLA Loneliness Scale used a four-point scale (“1 = never,” “2 = rarely,” “3 = sometimes,” or “4 = always”). While we asked the question using the original response categories in the 2021 survey, the 2020 survey used a five-point scale (“never,” “rarely,” “sometimes,” “often,” or “always”). Therefore, we combined the “often” and “always” categories after checking the distribution of responses (i.e., “1 = never,” “2 = rarely,” “3 = sometimes,” or “4 = often/always”). The possible scores ranged from 3 to 12, with a higher score indicating more severe loneliness. In this study, Cronbach's alphas, which were calculated using the three items of the scale, were 0.93 and 0.92 in 2020 and 2021, respectively.

#### Demographics, socioeconomic status, and health conditions

Sex, age, marital status (married or unmarried), and household composition (living alone or cohabiting) were included as demographics. Educational attainment (junior high school graduate, high school graduate, junior/vocational college graduate, university/graduate school graduate, or unknown/undisclosed), annual household income (≤2.9, 3.0–4.9, 5.0–6.9, 7.0–9.9, ≥10.0 million yen, or unknown/undisclosed), working status, and house ownership (yes or no) were asked as socioeconomic status. We used multiple indicators for working status because several studies reported that the COVID-19 outbreak affected mental health conditions as well as working situations among hospitality industry and medical/welfare workers (25–28): employment conditions [self-employed, permanent employment, temporary employment, or unemployed (including students)], job type (restaurant business, lodging business, medical service, welfare service, or others). In addition, participants were asked about the presence of chronic diseases [hypertension, diabetes, asthma, pneumonia, heart disease, stroke, chronic obstructive pulmonary disease (COPD), cancer, depression, and mental illness (other than depression)] as health conditions.

#### Prefectural COVID-19 outbreak situation

The cumulative number of confirmed cases per 100,000 population in the residential prefecture from January 15, 2020 (the day the first case of COVID-19 was confirmed in Japan) to September 30, 2021, was calculated, and the participants were divided into four groups based on quartiles. We used the following four categories: highest (≥1162.22 cases per 100,000 population), second highest (713.03–1162.21 cases per 100,000 population), second lowest (461.30–713.02 cases per 100,000 population), or lowest (≤461.29 cases per 100,000 population).

### Statistical analyses

We estimated the prevalence of social isolation (population proportion) and loneliness (population mean) using 95% confidence intervals (CIs) in the 2020 and 2021 surveys, respectively. In addition, we estimated the difference in prevalence between 2020 and 2021 with a 95% CI. These were calculated for the total sample and the stratified groups by demographics, socioeconomic status, and health conditions. Furthermore, we illustrated the prevalence by residential prefectures according to the COVID-19 outbreak. In the estimations, we used generalized estimating equations that considered the extra component of variation within participants in order to adjust for correlation among repeated measures. The analysis was performed using IBM SPSS Statistics 29 (IBM Corp., Armonk, NY, USA).

To describe the prevalence of social isolation and loneliness, we used sampling weights that were calculated based on logistic regression analysis using sex, age, and socioeconomic factors to adjust for differences between the respondents of the present web-based survey and the respondents in a widely used population-based sample representative of the Japanese population from the 2016 Comprehensive Survey of Living Conditions (29). The weighting was expected to adjust for a biased demographic distribution.

In addition, the age distributions of social isolation and loneliness were visualized with a weighted scatterplot smoothing spline curve fitted to the bivariate distributions of age and social isolation/loneliness in 2020 and 2021, respectively. Both the mean lines and smoothing spline curves were plotted with 95% confidence bands. We used the SAS PROC SGPLOT procedure (SAS Institute Inc., Cary, NC, USA).

## Results

Table 1 presents the weighted prevalence of social isolation and loneliness among the participants. The proportion (95% CI) of individuals with social isolation in the total sample was 27.4% (25.9, 28.9) in 2020 and 22.7% (21.9, 23.5) in 2021. The percentage difference from 2020 to 2021 was −4.7 percentage points (−6.3, −3.1), indicating that social isolation decreased during the interval. Concerning loneliness, the weighted mean score of the UCLA Loneliness Scale was 5.03 (4.86, 5.20) in 2020 and 5.86 (5.81, 5.91) in 2021, and the score difference was 0.83 points (0.66, 1.00). This suggests that, contrary to social isolation, loneliness increased from 2020 to 2021.

**Table 1:** The weighted prevalence of social isolation and loneliness by sex and age.

		**Distribution**		**Social isolation (possible proportion range: 0–100%)**			**Loneliness (possible score range: 3–12)**		
		**2020 %**	**2021 %**	**2020** **% (95% CI)**	**2021** **% (95% CI)**	**Difference percentage point (95% CI)**	**2020 mean (95% CI)**	**2021 mean (95% CI)**	**Difference mean (95% CI)**
Total		100.0%	100.0%	27.4 (25.9, 28.9)	22.7 (21.9, 23.5)	−4.7^***^ (−6.3, −3.1)	5.03 (4.86, 5.20)	5.86 (5.81, 5.91)	0.83^***^ (0.66, 1.00)
Sex	Men	49.7%	49.2%	34.0 (31.7, 36.5)	29.7 (28.5, 30.9)	−4.4^**^ (−7.0, −1.8)	5.01 (4.71, 5.30)	5.62 (5.55, 5.68)	0.61^***^ (0.31, 0.91)
	Women	50.3%	50.8%	20.8 (18.9, 22.8)	15.9 (15.0, 16.9)	−4.9^***^ (−7.0, −2.8)	5.05 (4.90, 5.21)	6.09 (6.02, 6.16)	1.04^***^ (0.88, 1.20)
Age	15–19 years	4.8%	2.0%	22.8 (16.9, 29.9)	7.1 (4.7, 10.6)	−15.7^***^ (−22.8, −8.5)	5.58 (5.09, 6.06)	6.06 (5.74, 6.38)	0.48 (−0.09, 1.05)
	20–29 years	12.6%	12.9%	15.0 (11.6, 19.3)	17.2 (15.4, 19.3)	2.2 (−2.0, 6.4)	7.01 (6.18, 7.83)	6.13 (5.98, 6.28)	−0.88^*^ (−1.72, −0.05)
	30–39 years	14.8%	14.7%	27.0 (25.0, 29.1)	24.8 (22.8, 26.8)	−2.2 (−4.9, 0.5)	5.34 (5.21, 5.48)	5.99 (5.87, 6.11)	0.64^***^ (0.48, 0.81)
	40–49 years	19.2%	19.4%	31.2 (29.5, 33.0)	26.0 (24.4, 27.7)	−5.2^***^ (−7.4, −3.1)	5.08 (4.98, 5.18)	6.09 (5.99, 6.19)	1.01^***^ (0.88, 1.13)
	50–59 years	16.7%	17.0%	31.8 (29.9, 33.7)	24.5 (22.9, 26.1)	−7.3^***^ (−9.6, −5.0)	4.80 (4.70, 4.91)	6.14 (6.04, 6.24)	1.33^***^ (1.21, 1.46)
	60–69 years	16.7%	17.3%	30.0 (27.7, 32.5)	23.5 (21.4, 25.8)	−6.5^***^ (−9.5, −3.6)	4.22 (4.11, 4.34)	5.47 (5.35, 5.59)	1.25^***^ (1.10, 1.39)
	70–79 years	15.3%	16.7%	26.9 (20.9, 33.9)	20.5 (18.4, 22.8)	−6.4 (−13.1, 0.2)	3.99 (3.56, 4.41)	5.36 (5.25, 5.48)	1.38^***^ (0.95, 1.80)

CI, Confidence interval. Asterisks indicate statistically significant differences between 2020 and 2021: ^***^p < 0.001, ^**^p < 0.01, ^*^p < 0.05.

The weighted prevalence of social isolation and loneliness, according to sex and age, is shown in Table 1. Social isolation was significantly greater in men than in women both in 2020 and 2021, but the decrease was similar for men and women [men: −4.4 percentage points (−7.0, −1.8); women: −4.9 percentage points (−7.0, −2.8)]. The level of loneliness was not different by sex in 2020, but in 2021, women had greater loneliness than men: 5.01 (4.71, 5.30) in men and 5.05 (4.90, 5.21) in women in 2020, and 5.62 (5.55, 5.68) in men and 6.09 (6.02, 6.16) in women in 2021. The level of increase in loneliness tended to be greater in women than men [men: 0.61 points (0.31, 0.91); women: 1.04 points (0.88, 1.20)].

The proportion of social isolation in younger ages, particularly 20–29 years, was lower than that in other age subgroups in both 2020 and 2021. The decrease was greatest in the 15–19 years age group [−15.7 percentage points (−22.8, −8.5)]. The UCLA Loneliness Scale score was the highest in the subgroup aged 20–29 years in 2020 [7.01 (6.18, 7.83)], but the score decreased to 6.13 (5.98, 6.28) in 2021. In the other age subgroups, the score increased from 2020 to 2021, particularly among the middle-aged and older populations.

**Figure 1:**
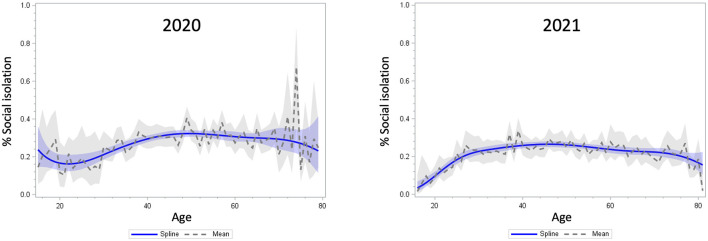
Distribution of the proportion of social isolation by age in 2020 **(left)** and 2021 **(right)**. The confidence bands reflects the 95% confidence intervals of the lines.

**Figure 2:**
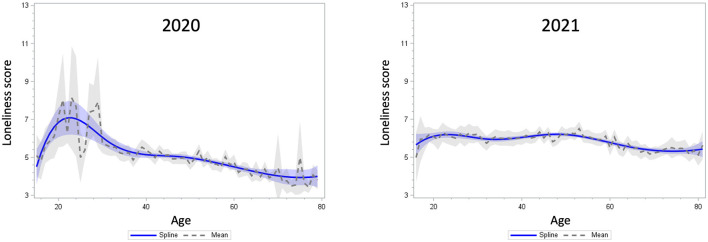
Distribution of the UCLA loneliness scale scores by age in 2020 **(left)** and 2021 **(right)**. The confidence bands reflects the 95% confidence intervals of the lines.

Figures 1, 2 display the observed distribution of social isolation and loneliness across age groups (solid lines). Regarding social isolation, the age distribution in 2020 was characterized by a dip around age 20 in early life; however, in the period of middle and old ages, the distribution was almost stable. In 2021, although the proportion of social isolation decreased among those under the age of 20 compared to 2020, the overall trend after the age of 20 was similar to the 2020 distribution. In contrast, the age distribution of loneliness in 2020 was characterized by a sharp peak around the age of 20 and a downward trend in middle and late adulthood. However, as indicated by the broad confidence band, there was large inter-individual variability in loneliness in this age group. After 1 year, the level of loneliness flattened across young and middle adulthood and declined continuously into old age.

Table 2 represents the weighted prevalence of social isolation and loneliness by demographics and socioeconomic status. Those who were married had a greater proportion of social isolation in 2020, but it significantly decreased in 2021 compared to unmarried people. Loneliness was consistently greater in unmarried individuals than in married individuals in both years. In 2020, people cohabiting had a higher proportion of social isolation than those living alone; however, this disparity was attenuated by 2021. Loneliness was greater among those living alone than among those living with others in both 2020 and 2021.

**Table 2:** The weighted prevalence of social isolation and loneliness by demographics and socioeconomic status.

		**Distribution**		**Social isolation (possible proportion range: 0–100%)**			**Loneliness (possible score range: 3–12)**		
		**2020 %**	**2021 %**	**2020** **% (95% CI)**	**2021** **% (95% CI)**	**Difference percentage point (95% CI)**	**2020 mean (95% CI)**	**2021 mean (95% CI)**	**Difference mean (95% CI)**
Marital status	Married	63.2%	64.8%	28.1 (26.5, 29.9)	21.6 (20.6, 22.6)	−6.6^***^ (−8.4, −4.7)	4.59 (4.48, 4.70)	5.60 (5.54, 5.66)	1.01^***^ (0.90, 1.13)
	Unmarried	36.8%	35.2%	26.1 (23.5, 28.9)	24.8 (23.5, 26.0)	−1.3 (−4.3, 1.6)	5.79 (5.42, 6.17)	6.34 (6.26, 6.42)	0.55^**^ (0.17, 0.92)
Household composition	Living alone	16.2%	15.8%	21.9 (18.3, 26.0)	22.4 (20.5, 24.3)	0.5 (−3.6, 4.6)	5.46 (4.82, 6.09)	6.26 (6.15, 6.37)	0.80^*^ (0.16, 1.44)
	Cohabiting	83.8%	84.2%	28.4 (26.9, 30.0)	22.8 (21.9, 23.6)	−5.7^***^ (−7.4, −4.0)	4.95 (4.79, 5.10)	5.78 (5.73, 5.84)	0.84^***^ (0.68, 0.99)
Education	Junior high school graduate	6.8%	4.0%	36.7 (29.8, 44.3)	32.5 (25.5, 40.4)	−4.2 (−14.2, 5.8)	4.56 (4.21, 4.91)	5.71 (5.31, 6.11)	1.15^***^ (0.65, 1.65)
	High school graduate	37.8%	48.8%	31.4 (29.7, 33.2)	23.1 (22.0, 24.4)	−8.3^***^ (−10.2, −6.3)	4.88 (4.75, 5.02)	5.83 (5.75, 5.90)	0.94^***^ (0.80, 1.09)
	Junior/vocational college graduate	19.3%	20.1%	26.4 (24.8, 28.0)	19.9 (18.6, 21.2)	−6.5^***^ (−8.4, −4.6)	5.04 (4.93, 5.14)	5.97 (5.88, 6.06)	0.93^***^ (0.81, 1.06)
	University/graduate school graduate	35.2%	25.8%	21.6 (18.7, 24.8)	21.9 (20.9, 22.9)	0.3 (−2.9, 3.4)	5.28 (4.85, 5.71)	5.87 (5.80, 5.93)	0.59^**^ (0.16, 1.01)
	Unknown/ undisclosed	0.8%	1.2%	36.2 (19.3, 57.3)	36.5 (24.7, 50.1)	0.3 (−23.4, 23.9)	4.75 (3.85, 5.66)	5.60 (5.02, 6.19)	0.85 (−0.24, 1.94)
Annual household income	≤2.9 million yen	18.5%	17.9%	31.6 (27.9, 35.6)	27.0 (24.9, 29.1)	−4.7^*^ (−8.9, −0.5)	5.31 (4.92, 5.70)	6.29 (6.17, 6.41)	0.98^***^ (0.58, 1.38)
	3.0–4.9 million yen	21.6%	21.4%	27.5 (24.3, 31.1)	21.7 (20.1, 23.3)	−5.9^**^ (−9.6, −2.2)	5.21 (4.80, 5.62)	5.93 (5.83, 6.04)	0.72^***^ (0.31, 1.14)
	5.0–6.9 million yen	15.5%	15.5%	24.0 (21.6, 26.6)	21.9 (20.0, 23.9)	−2.1 (−5.2, 1.0)	4.79 (4.62, 4.95)	5.70 (5.58, 5.82)	0.91^***^ (0.72, 1.11)
	7.0–9.9 million yen	14.2%	14.3%	23.1 (19.8, 26.9)	19.5 (17.8, 21.3)	−3.6 (−7.5, 0.3)	5.21 (4.54, 5.88)	5.59 (5.48, 5.71)	0.38 (−0.30, 1.06)
	≥10.0 million yen	8.2%	7.6%	15.7 (12.7, 19.3)	16.6 (14.5, 18.9)	0.9 (−3.0, 4.7)	4.75 (4.19, 5.31)	5.42 (5.27, 5.58)	0.67^*^ (0.10, 1.25)
	Unknown/ undisclosed	22.0%	23.3%	33.2 (29.8, 36.7)	24.8 (23.2, 26.6)	−8.3^***^ (−12.1, −4.5)	4.77 (4.61, 4.94)	5.87 (5.76, 5.97)	1.09^***^ (0.91, 1.27)
Employment condition	Self-employed	11.8%	10.0%	22.3 (17.9, 27.4)	20.6 (18.3, 23.0)	−1.7 (−7.0, 3.6)	5.75 (4.92, 6.58)	5.60 (5.46, 5.74)	−0.15 (−0.98, 0.69)
	Permanent employment	30.9%	32.7%	29.3 (27.4, 31.2)	23.5 (22.3, 24.7)	−5.8^***^ (−8.0, −3.6)	4.97 (4.73, 5.21)	5.72 (5.65, 5.80)	0.75^***^ (0.50, 1.00)
	Temporary employment	19.1%	18.9%	27.1 (23.5, 30.9)	20.8 (19.1, 22.6)	−6.3^**^ (−10.2, −2.3)	5.15 (4.76, 5.54)	5.99 (5.88, 6.10)	0.84^***^ (0.44, 1.24)
	Student	5.6%	3.7%	17.1 (12.3, 23.3)	8.9 (7.0, 11.4)	−8.2^**^ (−14.1, −2.3)	5.36 (5.03, 5.68)	5.97 (5.74, 6.21)	0.62^**^ (0.22, 1.01)
	Unemployed	32.5%	34.7%	29.4 (26.6, 32.3)	25.1 (23.6, 26.6)	−4.3^**^ (−7.3, −1.3)	4.70 (4.52, 4.88)	5.98 (5.89, 6.07)	1.28^***^ (1.09, 1.46)
Job type	Restaurant business	2.0%	2.7%	25.4 (20.0, 31.6)	15.2 (11.5, 19.8)	−10.2^**^ (−16.9, −3.5)	5.68 (5.26, 6.11)	5.81 (5.52, 6.11)	0.13 (−0.35, 0.61)
	Lodging business	0.5%	0.5%	17.8 (11.2, 27.3)	16.3 (9.5, 26.4)	−1.6 (−12.8, 9.7)	5.15 (4.50, 5.80)	5.71 (5.14, 6.29)	0.56 (−0.28, 1.40)
	Medical service	4.7%	4.1%	19.2 (14.0, 25.7)	13.9 (11.6, 16.6)	−5.3 (−11.4, 0.9)	5.86 (4.56, 7.15)	5.93 (5.71, 6.15)	0.07 (−1.24, 1.38)
	Welfare service	2.7%	3.0%	24.0 (19.4, 29.3)	21.4 (16.8, 26.9)	−2.6 (−9.0, 3.9)	5.40 (4.99, 5.81)	5.83 (5.57, 6.09)	0.43 (−0.03, 0.88)
	Others	90.1%	89.7%	28.0 (26.4, 29.7)	23.4 (22.6, 24.2)	−4.6^***^ (−6.4, −2.9)	4.96 (4.79, 5.13)	5.86 (5.81, 5.91)	0.90^***^ (0.73, 1.07)
House ownership	Yes	73.9%	76.2%	28.6 (27.0, 30.3)	22.7 (21.8, 23.6)	−6.0^***^ (−7.8, −4.2)	4.79 (4.66, 4.92)	5.77 (5.72, 5.83)	0.98^***^ (0.84, 1.12)
	No	26.1%	23.8%	23.9 (21.0, 27.2)	22.8 (21.4, 24.2)	−1.1 (−4.4, 2.1)	5.70 (5.24, 6.17)	6.13 (6.04, 6.23)	0.43 (−0.04, 0.90)

CI: Confidence interval. Asterisks indicate statistically significant differences between 2020 and 2021: ^***^p < 0.001, ^**^p < 0.01, ^*^p < 0.05.

People with lower education were more likely to be socially isolated but have lower loneliness in 2020, whereas people with lower income tended to be socially isolated and have higher loneliness. These trends continued in 2021. Concerning employment conditions, those with permanent jobs and those who were unemployed (including homemakers) had a higher proportion of social isolation by 2020. These decreased in 2021, but the proportions remained higher than those in the other categories. Social isolation among students remarkably improved in 2021 compared to 2020. The disparity in loneliness by employment condition observed in 2020 was suppressed by 2021. Social isolation among restaurant workers was high in 2020 but decreased in 2021.

Finally, Table 3 illustrates the weighted prevalence of social isolation and loneliness by health conditions and outbreak situation in the residential prefecture. People with each chronic disease were less likely to be socially isolated, but they experienced higher loneliness in 2020. The changing trend of social isolation varied according to the type of chronic disease, and there was a general decrease in loneliness in 2021. Changes in the proportion of social isolation and loneliness scores were smaller in prefectures with greater numbers of COVID-19 infection cases.

**Table 3:** The weighted prevalence of social isolation and loneliness by health conditions and prefectural in the COVID-19 outbreak situation.

		**Distribution**		**Social isolation (possible proportion range: 0–100%)**			**Loneliness (possible score range: 3–12)**		
		**2020 %**	**2021 %**	**2020** **% (95% CI)**	**2021** **% (95% CI)**	**Difference percentage point (95% CI)**	**2020 mean (95% CI)**	**2021 mean (95% CI)**	**Difference mean (95% CI)**
Hypertension	Not possessing	80.2%	74.5%	26.6 (25.1, 28.2)	22.8 (21.9, 23.7)	−3.8^***^ (−5.6, −2.1)	5.08 (4.90, 5.25)	5.87 (5.81, 5.92)	0.79^***^ (0.61, 0.97)
	Possessing	19.8%	25.5%	30.5 (26.6, 34.6)	22.4 (20.7, 24.1)	−8.1^***^ (−12.3, −3.9)	4.84 (4.41, 5.27)	5.83 (5.73, 5.93)	1.00^***^ (0.56, 1.43)
Diabetes	Not possessing	92.6%	92.0%	27.2 (25.7, 28.7)	22.7 (21.9, 23.5)	−4.5^***^ (−6.2, −2.9)	5.01 (4.85, 5.17)	5.88 (5.83, 5.93)	0.87^***^ (0.71, 1.03)
	Possessing	7.4%	8.0%	29.8 (23.3, 37.1)	22.8 (19.9, 26.0)	−6.9 (−14.2, 0.3)	5.28 (4.26, 6.31)	5.63 (5.46, 5.79)	0.34 (−0.69, 1.37)
Asthma	Not possessing	94.2%	96.0%	28.1 (26.6, 29.6)	22.9 (22.1, 23.7)	−5.2^***^ (−6.8, −3.6)	4.89 (4.76, 5.02)	5.84 (5.79, 5.89)	0.95^***^ (0.82, 1.09)
	Possessing	5.8%	4.0%	16.1 (10.4, 24.2)	17.7 (14.3, 21.8)	1.6 (−6.0, 9.2)	7.37 (6.09, 8.66)	6.34 (6.10, 6.59)	−1.03 (−2.33, 0.27)
Pneumonia	Not possessing	97.2%	97.7%	27.7 (26.3, 29.2)	22.9 (22.2, 23.7)	−4.8^***^ (−6.4, −3.2)	4.93 (4.79, 5.08)	5.85 (5.80, 5.90)	0.92^***^ (0.77, 1.07)
	Possessing	2.8%	2.3%	15.1 (6.9, 29.8)	12.4 (8.3, 18.0)	−2.7 (−14.9, 9.5)	8.43 (7.08, 9.78)	6.21 (5.89, 6.53)	−2.22^**^ (−3.57, −0.87)
Heart disease	Not possessing	95.9%	97.1%	28.0 (26.5, 29.4)	22.8 (22.0, 23.6)	−5.2^***^ (−6.8, −3.6)	4.92 (4.79, 5.06)	5.85 (5.80, 5.90)	0.93^***^ (0.79, 1.07)
	Possessing	4.1%	2.9%	14.1 (7.9, 24.0)	20.3 (15.9, 25.6)	6.2 (−3.0, 15.4)	7.60 (6.14, 9.06)	6.18 (5.89, 6.47)	−1.42 (−2.89, 0.06)
Stroke	Not possessing	98.2%	98.2%	27.6 (26.2, 29.1)	22.9 (22.1, 23.7)	−4.8^***^ (−6.4, −3.2)	4.97 (4.82, 5.12)	5.85 (5.80, 5.90)	0.88^***^ (0.73, 1.03)
	Possessing	1.8%	1.8%	13.7 (5.3, 31.0)	13.8 (8.7, 21.4)	0.1 (−13.9, 14.1)	8.32 (6.26, 10.37)	6.39 (6.05, 6.73)	−1.92 (−3.98, 0.13)
COPD	Not possessing	98.2%	98.5%	27.7 (26.2, 29.2)	22.8 (22.1, 23.6)	−4.8^***^ (−6.4, −3.2)	4.97 (4.82, 5.12)	5.85 (5.81, 5.90)	0.88^***^ (0.73, 1.04)
	Possessing	1.8%	1.5%	12.5 (4.3, 31.0)	12.9 (7.6, 20.9)	0.4 (−13.7, 14.5)	8.18 (6.01, 10.36)	6.17 (5.76, 6.59)	−2.01 (−4.21, 0.20)
Cancer	Not possessing	96.5%	97.4%	27.9 (26.5, 29.4)	23.0 (22.2, 23.8)	−4.9^***^ (−6.5, −3.4)	4.92 (4.79, 5.05)	5.86 (5.81, 5.91)	0.94^***^ (0.80, 1.07)
	Possessing	3.5%	2.6%	12.8 (6.8, 23.0)	12.5 (9.4, 16.5)	−0.3 (−8.9, 8.2)	8.07 (6.65, 9.49)	5.95 (5.69, 6.21)	−2.12^**^ (−3.56, −0.68)
Depression	Not possessing	95.0%	95.4%	27.8 (26.4, 29.3)	22.5 (21.7, 23.3)	−5.4^***^ (−7.0, −3.8)	4.83 (4.69, 4.97)	5.75 (5.70, 5.80)	0.92^***^ (0.78, 1.06)
	Possessing	5.0%	4.6%	18.8 (12.4, 27.4)	27.2 (23.7, 31.1)	8.4^*^ (0.4, 16.5)	8.78 (7.81, 9.75)	8.12 (7.91, 8.33)	−0.66 (−1.64, 0.32)
Mental illness (other than depression)	Not possessing	94.9%	95.6%	27.8 (26.4, 29.3)	22.7 (21.9, 23.5)	−5.1^***^ (−6.7, −3.5)	4.83 (4.70, 4.97)	5.77 (5.72, 5.82)	0.94^***^ (0.80, 1.08)
	Possessing	5.1%	4.4%	19.2 (12.5, 28.2)	22.8 (19.6, 26.4)	3.6 (−4.8, 12.1)	8.66 (7.86, 9.47)	7.78 (7.53, 8.04)	−0.88^*^ (−1.72, −0.05)
Prefectural COVID-19 outbreak situation	Highest	41.0%	46.6%	24.8 (22.3, 27.5)	24.9 (23.8, 25.9)	0.0 (−2.7, 2.8)	5.20 (4.85, 5.56)	5.77 (5.71, 5.83)	0.57^**^ (0.21, 0.93)
	Second highest	23.5%	21.8%	29.2 (26.5, 32.0)	22.0 (20.3, 23.8)	−7.2^***^ (−10.3, −4.1)	4.99 (4.79, 5.20)	5.96 (5.85, 6.07)	0.97^***^ (0.75, 1.12)
	Second lowest	18.0%	16.4%	29.9 (27.3, 32.6)	20.2 (18.3, 22.3)	−9.6^***^ (−12.8, −6.5)	5.00 (4.75, 5.26)	5.98 (5.85, 6.11)	0.98^***^ (0.72, 1.24)
	Lowest	17.5%	15.1%	28.5 (25.3, 31.9)	19.7 (17.7, 21.8)	−8.8^***^ (−12.4, −5.2)	4.71 (4.55, 4.87)	5.85 (5.70, 5.99)	1.14^***^ (0.95, 1.33)

CI, Confidence interval; COPD, chronic obstructive pulmonary disease; COVID-19, Coronavirus disease 2019.

Asterisks indicate statistically significant differences between 2020 and 2021: ^***^p < 0.001, ^**^p < 0.01, ^*^p < 0.05.

## Discussion

This study investigated changes in the prevalence of social isolation and loneliness among Japanese people between the first and second years of the COVID-19 outbreak. The study also examined the differences among subgroups defined by demographic characteristics, socioeconomic status, health conditions, and outbreak situations in residential prefectures. Overall, we found that social isolation decreased from 2020 to 2021, whereas loneliness increased.

In the first year of the pandemic in Japan, most schools were closed, and students received classes online. The extracurricular activity was also assessed. Many companies have introduced teleworking, including, as of April through May 2020, 56.4% of all companies, 83.3% of medium-sized companies, and 50.5% of small-sized companies (30). Additionally, most community activities were forced to refrain. In contrast, in the second year of the pandemic, the situation regarding COVID-19 had changed. For example, COVID-19 vaccination has been widespread, and people have understood and adopted infection-prevention behaviors naturally. Schools and community activities were reopened. An increasing number of workers were going to the office; the proportion of teleworkers was 38.4% in March 2021 (30). Under these circumstances, the COVID-19 outbreak struck everyone's social connections with others and society; however, the influence was relieved in the second year. Therefore, social isolation decreased by September and October of 2021 compared to 2020.

However, there were variations in the degree of change according to participants' characteristics. The decrease was particularly large for those aged 15–19 years, which is likely due to the school's shift to face-to-face classes in 2021. The proportion of social isolation among the older population was higher than that among the younger population as of 2020, but the percentages decreased more sharply among those in the older groups than among those in the younger groups by September and October of 2021. As previous research indicated (7), older people in Japan were not familiar with online communication tools such as the Internet (e.g., Zoom and Skype) and social networking services (SNSs) (31). Another survey showed that the proportion of older people who reported an increase in the frequency of use of online connections was lower in Japan than in Western countries, including the United States, Germany, and Sweden (32). Thus, social isolation tended to be prevalent in the older population during the first year of the pandemic. However, because community activities, the major participants of which were older people (33), have been reopened, they could be less socially isolated in the second year.

People who were married and cohabiting were more likely to be socially isolated than their counterparts in the first year of the pandemic. A previous study conducted before the COVID-19 pandemic reported that older people living with others tended to become more socially isolated than those living alone (21). People who were married or living with someone tended to complete their social interactions within the household without having connections with those outside the household, particularly during the pandemic period. However, these gaps observed in 2020 were attenuated in 2021, which implies that people expanded their daily activities in the second year of the pandemic when restrictions on people's behaviors eased.

The proportion of social isolation has decreased significantly among restaurant workers. In the first year of the outbreak, most prefectural governments in Japan requested shorter hours or restaurant closures. Contrarily, in the second year, prefectures (except those with large numbers of COVID-19 cases) did not. Therefore, a remarkable decline in the proportion of restaurant workers is observed. In the prefectures with fewer COVID-19 infection cases, we observed a greater decrease in social isolation. In such prefectures, municipal governments tend to request fewer behavioral restrictions on people, especially in the second year. People in the prefectures were somewhat free of social activities; thus, they were unlikely to be socially isolated.

In contrast to social isolation, loneliness increased in almost all the strata. This implies that the prolonged pandemic may have caused people to feel overwhelmed and exhausted, which is sometimes called “COVID-19-related psychological fatigue” (34), resulting in an increase in loneliness from 2020 to 2021. By age group, loneliness was higher among younger generations in 2020, especially among those aged 15–29 years, although they were not likely to be socially isolated. This result is consistent with those of a public cross-sectional survey conducted in Japan (35). Social isolation and loneliness are similar concepts but not the same. While the younger generation is good at connecting through the Internet and SNSs, such online connections are less likely to generate support that can address function limitation concerns (36, 37). Therefore, they might feel lonely, even if they have more online connections. In addition, people who were unmarried and living alone had greater loneliness during the study period, although the proportions of social isolation among them were lower in 2020. Earlier research presented a similar trend (38, 39). These similar findings provide evidence to support that social isolation and loneliness are not necessarily the same concept.

As an exception, loneliness was higher among those with chronic illness than their counterparts in the first year, and it decreased in the second year. A study in Denmark reported a similar trend (17). Psychological burdens such as anxiety and worries about COVID-19 may have decreased with the gradual establishment of infection-prevention behaviors in the population and with the spread of COVID-19 vaccination (17). Those with chronic illness and at a higher risk of COVID-19 infection might have carried a greater psychological burden in 2020 due to alienation from others, but this condition may have eased by 2021. Loneliness was higher in prefectures with more infections in the first year. However, in the second year, it increased in all prefectures, particularly within those with fewer infection cases. As a result, the disparity among the prefectures was reduced, suggesting that loneliness was pervasive, regardless of infection status.

Some limitations must be considered. First, because we used data from the web-based survey, the sample did not necessarily reflect the demographic distribution of the general population. This potential selection bias was likely to result in more optimistic estimates than the true levels in the population. Fundamentally, average social isolation and loneliness levels are likely to be worse than those reported in this study. Considering this, a weighting technique using external nationally representative data was applied to reduce bias. However, a residual bias may still exist. For example, people who were less negatively affected by the pandemic tended to answer online surveys. Furthermore, older people with better cognitive function and technological competencies were more likely to participate in the survey than those with worse status and those lacking technological competencies. Second, questions regarding social isolation did not include work-related connections, although we measured the frequency of contact with family members or relatives who were living apart and friends/neighbors. Thus, the prevalence of social isolation, particularly in young and middle-aged individuals, may have been underestimated. Various forms of social connections should be assessed in future surveys to capture social isolation conditions correctly. Third, this study only investigated participants' experiences at two time points during/after the pandemic. The level of social isolation and loneliness could be influenced by social events of the moment (e.g., infection spread, lockdown, stay-at-home order) (17). A time trend study is necessary to capture the status of these indicators accurately. Finally, the results included some response categories with small proportions of participants (e.g., job types and health conditions). We deemed that the prevalence of social isolation and loneliness in these categories could be useful information to understand vulnerable populations that were strongly influenced by the COVID-19 pandemic. However, because a small sample size possibly leads to low statistical power, the results must be interpreted with caution.

## Conclusion

Social isolation decreased from the first year (2020) to the second year (2021) of the COVID-19 pandemic, whereas loneliness increased among the Japanese population. However, the trends in these changes differed according to the characteristics of the participants. Furthermore, the assessment of the impact of the COVID-19 pandemic on social isolation and loneliness will lead to an understanding of who was vulnerable during the pandemic. Based on the findings, the government and public health sector can take measures to prioritize those most affected by social isolation and loneliness and establish efficient strategies to prevent mental disorders in future pandemics.

## Data availability statement

The raw data supporting the conclusions of this article will be made available by the authors, without undue reservation.

## Ethics statement

The studies involving human participants were reviewed and approved by Research Ethics Committee of the Osaka International Cancer Institute. Written informed consent for participation was not required for this study in accordance with the national legislation and the institutional requirements.

## Author contributions

HM and TT designed the study and collected data. HM, TSu, IN, and TSh conducted data analyses. HM prepared the manuscript. TSu, IN, TSh, and TT provided critical feedback. All the authors have read and approved the final version of the manuscript.
