# Supplementary figures and images for: Changes in social isolation and loneliness prevalence during the COVID-19 pandemic in Japan: The JACSIS 2020–2021 study
Source: Front Public Health. 2023 Feb 16;11:1094340. doi: 10.3389/fpubh.2023.1094340 (PMC9978762; doi:10.3389/fpubh.2023.1094340)

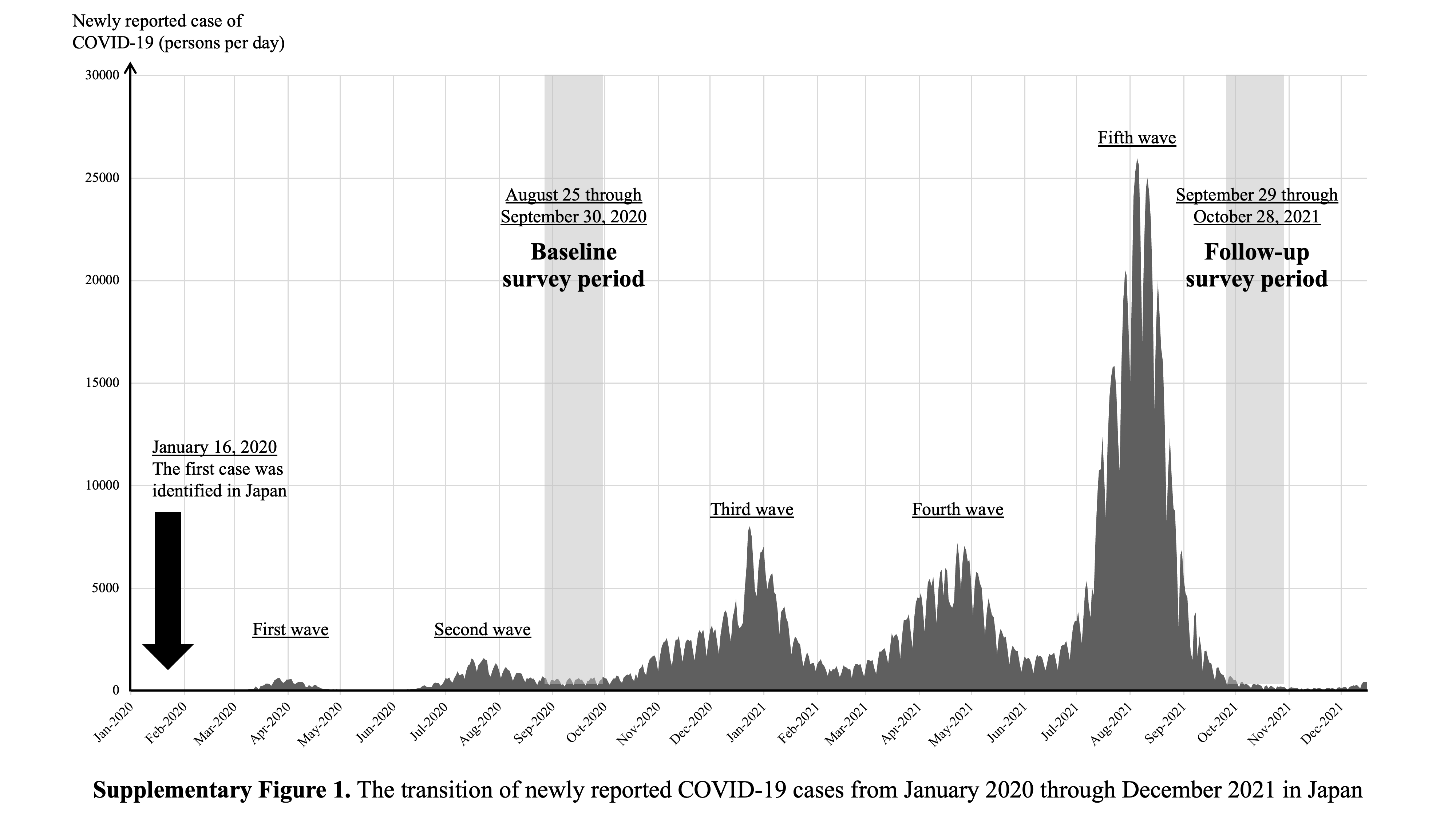

Supplement: Supplementary file 1 [file Image_1.TIFF]
